# A *Torilis japonica* Extract–GHK-Cu Complex Attenuates Th2 Cytokines and Promotes Keratinocyte Recovery: A Potential Antioxidant Strategy for Atopic Dermatitis

**DOI:** 10.3390/antiox15070818

**Published:** 2026-06-29

**Authors:** Soojin Jeon, Jihye Maeng, Jiwon Lee, Young-Min Kim, Gaewon Nam

**Affiliations:** 1 Department of Integrative Biotechnology, Yonsei University, 50 Yonsei-ro, Seodaemun-gu, Seoul 03722, Republic of Korea; wjstnwls511@yonsei.ac.kr (S.J.); jhmaeng701@yonsei.ac.kr (J.M.); won0817@yonsei.ac.kr (J.L.); 2 Department of Biological Science and Biotechnology, College of Life Science and Nano Technology, Hannam University, Daejeon 34054, Republic of Korea; kym@hnu.kr; 3 Bio-Living Engineering Major, Global Leaders College, Yonsei University, 50 Yonsei-ro, Seodaemun-gu, Seoul 03722, Republic of Korea

**Keywords:** atopic dermatitis, *Torilis japonica* extract, copper-tripeptide-1, GHK-Cu, Th2 cytokines, skin inflammation, keratinocyte regeneration, antioxidant activity

## Abstract

Atopic dermatitis (AD) is a chronic skin disorder driven by Th2 immune dysregulation, persistent inflammation, and epidermal barrier defects. Oxidative stress acts as a major upstream factor in this process, amplifying inflammatory signals and worsening disease severity. While current treatments relieve acute symptoms, long-term application is often constrained by side effects and poor barrier restoration, pointing to a need for safer, multifaceted alternatives. Here, we formulated a complex of *Torilis japonica* extract (TJE) and GHK-Cu (Glycyl-L-histidyl-L-lysine copper(II)) complex and examined its anti-atopic and skin-regenerative properties using a TNF-α (Tumor necrosis factor-α)/IFN-γ (Interferon-γ)-stimulated HaCaT cell model. TJE decreased the expression of AD-related chemokines (TARC(Thymus and activation-regulated chemokine (CCL17)) and CTACK(Cutaneous T-cell-attracting chemokine (CCL27)) as well as IgE production, confirming the suppression of Th2-driven inflammation. An optimized 6:4 ratio (TJE:GHK-Cu) yielded the highest efficacy compared to individual treatments, indicating a synergistic interaction. TJE–GHK-Cu complex suppressed the transcription of key Th2 cytokines (IL-4, IL-5, IL-10, and IL-13) and promoted keratinocyte migration during wound healing assays. The formulation also displayed strong radical scavenging activity without compromising cell viability. These results demonstrate that the TJE–GHK-Cu complex provides simultaneous anti-inflammatory, antioxidant, and regenerative benefits, presenting a formulation warranting further investigation for managing AD.

## 1. Introduction

Atopic dermatitis (AD) is a chronic, relapsing skin condition that is becoming increasingly common across all age groups [1]. Primary clinical symptoms include severe itching, erythema, dry skin, and barrier dysfunction, which severely impact patient quality of life [2]. The underlying pathology of AD is complex rather than relying on a single inflammatory pathway [3]. A defining feature is the overactivation of type 2 immune responses, characterized by the expansion of T helper 2 (Th2) cells and the subsequent release of specific cytokines such IL-4, IL-5, IL-10 and IL-13 [3]. This immunological shift leads to elevated serum IgE levels and mast cell activation, driving a continuous cycle of skin inflammation [3].

Alongside immune issues, compromised skin barrier integrity is a core mechanism of AD [4]. A healthy epidermal layer prevents excessive water loss and blocks environmental allergens through structural proteins like filaggrin, loricrin, and claudins [4,5,6]. AD patients typically show reduced levels of these proteins, making the skin more permeable to external irritants. This structural defect fuels further immune activation, trapping the patient in a feedback loop of barrier breakdown and inflammation [7].

Oxidative stress has also been identified as an important trigger in AD pathogenesis [3,4]. When keratinocytes produce excess reactive oxygen species (ROS), it aggravates inflammatory signaling and damages the epidermal barrier [3,7]. Specifically, high ROS levels can suppress the production of filaggrin and loricrin [5,6]. ROS also promotes Th2-dominant immune responses by activating redox-sensitive pathways, increasing the output of IL-4 and IL-13 [3,8]. Controlling oxidative stress therefore offers a practical way to manage inflammation and restore barrier function simultaneously. Keratinocytes themselves play an active role in this process by releasing epithelial cytokines like TSLP and IL-33 when exposed to inflammatory triggers [8,9]. These mediators boost the Th2 response, showing how epidermal and immune cells continuously interact to sustain AD lesions [10].

Standard AD treatments involve topical corticosteroids, immunomodulators, and biologic agents [11]. They generally control acute flare-ups well, but prolonged use carries risks such as skin thinning, immunosuppression, and symptom rebound [12]. Most conventional options focus solely on suppressing inflammation, offering little benefit for epidermal repair or cellular turnover [13]. As a result, researchers are increasingly looking into plant-derived materials like *Erigeron annuus* [14], *Glycine soja* [15], and *Nymphoides peltata* [16] extracts. While these natural derivatives have demonstrated anti-inflammatory properties, their translation to clinical use is hindered by poor skin penetration and low bioavailability in the deeper epidermal layers [17].

Peptides, conversely, are small bioactive molecules known for their high skin penetration, precise cellular targeting, and low immunogenicity [18,19]. In AD research, certain peptides have been shown to regulate inflammation, speed up epidermal repair, and restore the skin barrier all at once [20]. Recent animal and cell-based studies indicate that therapeutic peptides can reduce AD symptoms by blocking chemokines like (TARC(Thymus and activation-regulated chemokine (CCL17)) and CTACK(Cutaneous T-cell-attracting chemokine (CCL27))), directly interfering with key disease pathways [12].

GHK-Cu (copper tripeptide-1) is a natural copper-binding tripeptide (glycine-histidine-lysine) first isolated from human plasma in 1973 [21,22]. Its natural concentration drops with age, hinting at a role in physiological maintenance [21]. GHK-Cu is highly valued for tissue repair and skin regeneration [21]. It stimulates dermal fibroblasts to produce collagen and glycosaminoglycans, improving skin structure [22,23]. It also supports wound healing and hair follicle function [21], acting as a broad signaling molecule for extracellular matrix remodeling. However, GHK-Cu has practical limitations. Its hydrophilic nature restricts its ability to pass through the stratum corneum, limiting its delivery to the dermis [17,24]. The TJE–GHK-Cu complex is also sensitive to environmental pH and oxidative stress due to the redox potential of the copper ion. Furthermore, most supporting data comes from preclinical settings rather than extensive clinical trials [25,26].

*Torilis japonica* (TJ) is a traditional East Asian herb used to treat itching, eczema, and skin inflammation [27]. *Torilis japonica* extract (TJE) contains coumarin derivatives—such as osthole, imperatorin, and bergapten—that provide documented anti-inflammatory and antioxidant benefits [27,28,29,30]. Animal studies show that TJE can lower AD scores and reduce cell infiltration in DNCB-induced AD models [30]. Like many botanical extracts, though, TJE struggles with poor skin permeability [17,24].

To investigate the anti-atopic potential of TJE and GHK-Cu, we employed a TNF-α (Tumor necrosis factor-α)/IFN-γ (Interferon-γ)-stimulated HaCaT keratinocyte model. HaCaT cells are widely used in AD research because inflammatory stimulation induces the production of key AD-related mediators, including TARC (CCL17), CTACK (CCL27), and Th2-associated cytokines, thereby reproducing important inflammatory characteristics of AD. In addition, keratinocytes are central regulators of epidermal barrier function and cutaneous immune responses, making this model a useful platform for evaluating candidate therapeutic agents for AD [1,2,30].

Because both TJE and GHK-Cu face similar delivery and bioavailability challenges [17,24,31], combining them offers a logical solution. This study introduces a TJE–GHK-Cu complex designed to merge the botanical antioxidant properties of TJE with the regenerative signaling of GHK-Cu. The goal is to move beyond a simple mixture and achieve a synergistic effect that addresses inflammation, barrier disruption, and slow tissue turnover simultaneously. We evaluated TJE–GHK-Cu complex’s ability to inhibit AD-related chemokines and Th2 cytokines, promote keratinocyte wound healing, and maintain cellular safety, ultimately testing its viability as a comprehensive AD treatment.

## 2. Materials and Methods

### 2.1. Materials

Dried TJ fruits were cultivated in Yeongcheon, Gyeongsangbuk-do, Republic of Korea, and purchased from Joeun Yakcho (Yeongcheon, Republic of Korea). TJE was prepared via hot-water extraction using secondary purified distilled water at 120 °C and 1.5 atm for 2 h. The extract was freeze-dried into a powder and stored at room temperature.

GHK-Cu (glycyl-L-histidyl-L-lysine copper complex) was provided by Dr. Oracle (Seoul, Republic of Korea). A stock solution was prepared in sterile distilled water and diluted as required.

Other reagents included Dulbecco’s Modified Eagle’s Medium (DMEM; L0103-500, Biowest, Nuaillé, France), fetal bovine serum (FBS; S1480, Biowest, Nuaillé, France), and penicillin–streptomycin (Gibco, Thermo Fisher Scientific, Waltham, MA, USA); dimethyl sulfoxide (DMSO; Sigma-Aldrich, St. Louis, MO, USA); human keratinocytes (HaCaT, 300493, Cytion, Eppelheim, Germany) and human dermal fibroblasts (HS68, CRL-1635^™^, ATCC, Manassas, VA, USA); tumor necrosis factor-α (TNF-α) (210-TA, R&D systems, Minneapolis, MN, USA) and interferon-γ (IFN-γ) (285-IF, R&D Systems, Minneapolis, MN, USA), MTT assay kit (ab211091, Abcam, Cambridge, UK) and 2,2-diphenyl-1-picrylhydrazyl (DPPH; Sigma, St. Louis, MO, USA); enzyme-linked immunosorbent assay (ELISA) kits for thymus and activation-regulated chemokine (TARC) (DDN00, R&D Systems, Minneapolis, MN, USA), cutaneous T-cell attracting chemokine (CTACK) (DCC270, R&D Systems, Minneapolis, MN, USA), and human immunoglobulin E ELISA kit (IgE) (ab178659, Abcam, Cambridge, UK); RNA extraction kit (EZ™ Total RNA miniprep kit, IntronBiotech, EP301-50N, Seongnam, Republic of Korea) and complementary DNA (cDNA) synthesis kit (iScript cDNA synthesis kit; 1708890, Biorad, Hercules, CA, USA), and Real-Time PCR master mix (SsoAdvanced^™^ Universal SYBR^®^ Green Supermix; #1725270, Biorad, Hercules, CA, USA).

All unlisted chemicals were obtained from Sigma-Aldrich (St. Louis, MO, USA).

### 2.2. HPLC Analysis of TJE

The chemical profile of TJE was analyzed using high-performance liquid chromatography (HPLC, YMC J’sphere ODS-H80 column, YMC Co., Ltd., Kyoto, Japan). The analysis was performed using an Agilent 1200 series system (Agilent Technologies, Santa Clara, CA, USA) equipped with a YMC J’sphere ODS-H80 column (YMC Co., Ltd., Kyoto, Japan) (Table 1) The mobile phase consisted of 0.05% trifluoroacetic acid in distilled water (A) and methanol (B). The gradient condition was set from A:B = 80:20 at 0 min to 0:100 over 30 min. The flow rate was maintained at 1 mL/min, and the detection wavelength was set at 254 nm. For the analysis of purified active fractions, an Agilent 1200 series system equipped with an InertSustain C18 column (GL Sciences, Tokyo, Japan) (5 μm, 4.6 × 150 mm) was used. The mobile phase consisted of water and methanol, with a gradient from 20% methanol to 100% methanol over 8 min, followed by 100% methanol maintained until 13 min (Table 1).

### 2.3. Cell Culture

HaCaT and HS68 were cultured in DMEM containing 10% FBS and 1% penicillin-streptomycin. Cells were incubated at 37 °C in a humidified incubator (NU-5700, NUAIRE, Plymouth, MN, USA) with 5% CO_2_.

### 2.4. TNF-α/IFN-γ-Induced Inflammatory Model in HaCaT Cells

An in vitro AD model was established by treating HaCaT with 10 ng/mL of TNF-α/IFN-γ for 1 h. The cells were exposed to TJE, GHK-Cu, or the TJE–GHK-Cu complex based on the specific experimental conditions.

### 2.5. Measurement of TARC, CTACK, and IgE Levels

HaCaT cells were stimulated with TNF-α/IFN-γ to induce AD-like inflammatory conditions and then treated with TJE at concentrations ranging from 1 to 100 μg/mL for dose–response analysis. For the combination experiments, cells were treated with TJE at 20 or 30 μg/mL either alone or as TJE–GHK-Cu complexes prepared at the indicated TJE:GHK-Cu weight ratios of 8:2, 6:4, 4:6, and 2:8 (*w*/*w*). Cell culture supernatants were collected 24 h after treatment, centrifuged to remove cellular debris, and used for ELISA analysis. The extracellular levels of TARC, CTACK, and IgE were quantified using ELISA kits according to the manufacturers’ instructions. ELISA data were calculated using standard curves for each target analyte and expressed as concentrations in the culture supernatant; the values were not normalized to total protein concentration.

### 2.6. Analysis of IL-4, IL-5, IL-10 and IL-13 mRNA Expression

Following TNF-α/IFN-γ stimulation, HaCaT cells were treated with TJE alone at 20 or 30 μg/mL, or with TJE–GHK-Cu complexes prepared at the optimized TJE:GHK-Cu mixing ratio of 6:4 (*w*/*w*). These groups correspond to TJE20, TJE30, TJE20+P, and TJE30+P, respectively. Total RNA was extracted and reverse-transcribed into cDNA. The expression levels of IL-4, IL-5, IL-10, and IL-13 were analyzed using RT-qPCR. β-actin was used as the housekeeping gene for normalization, and relative gene expression levels were calculated using the 2^−ΔΔCt^ method. Primer sequences are listed in Table 2.

### 2.7. Wound Healing Analysis

HaCaT cells were seeded in 6-well plates (3.0 × 10^5^ cells/well) and grown to 100% confluence. After a 1 h pretreatment with TNF-α/IFN-γ to simulate AD inflammation, a standard scratch was made across the cell monolayer using a sterile 200 μL pipette tip (Axygen^®^, Corning Inc., Corning, NY, USA). Detached cells were washed away with PBS. The remaining cells were treated with TJE, the GHK-Cu, or TJE–GHK-Cu complex. Cell migration was recorded at 0 h and 48 h using an optical microscope (CKX53, Olympus Corporation, Tokyo, Japan) at × 200 magnification. The wound area was measured via ImageJ software (version 1.54p, National Institutes of Health, Bethesda, MD, USA), and the closure rate was calculated relative to the initial gap size.

### 2.8. Cytotoxicity of TJE

Cells were seeded in 96-well plates at a density of 9.5 × 10^5^ cells/mL. Following a 24 h incubation, cells were exposed to TJE (50–150 μg/mL), peptide, or TJE–GHK-Cu complex for another 24 h. Viability was measured by adding MTT reagent (MTT assay kit, ab211091, Abcam, Cambridge, UK), dissolving the formed formazan crystals in DMSO, and reading the absorbance at 570 nm with a microplate reader (Epoch SN, Biotek Instruments, Winooski, VT, USA).

### 2.9. DPPH Radical Scavenging Assay of TJE, Peptide, and TJE–GHK-Cu Complex

The antioxidant capacity of TJE, peptide, and the TJE–GHK-Cu complex were tested using the DPPH radical scavenging assay (Sigma-Aldrich, St. Louis, MO, USA). DPPH solution was mixed with the test samples at various concentrations and incubated in the dark at room temperature. Absorbance was recorded at 517 nm. Vitamin C and PBS served as positive and negative controls, respectively. Scavenging activity was calculated as the percentage decrease in absorbance compared to the control.

### 2.10. Data and Statistical Analysis

All assays were performed using technical triplicates for each experimental condition, unless otherwise indicated. Data are expressed as mean ± standard deviation (SD). Differences among groups were evaluated using one-way ANOVA followed by Tukey’s multiple-comparison test. Statistical significance was defined as *p* < 0.05. Unless otherwise indicated, * *p* < 0.05, ** *p* < 0.01, and *** *p* < 0.001 were used to denote statistical significance. The comparison groups are specified in each figure legend.

## 3. Results

### 3.1. HPLC Characterization of Torilis japonica

**Figure 1:**
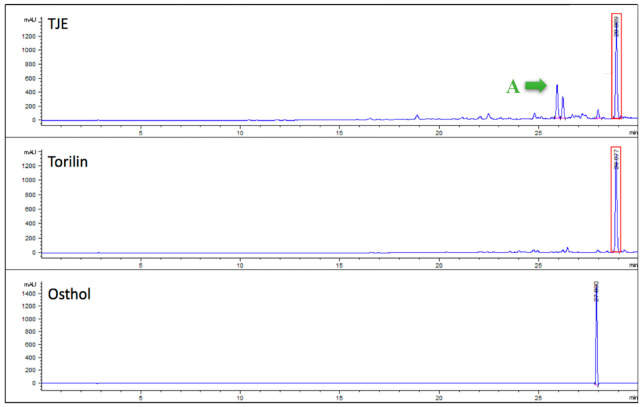
HPLC fingerprint analysis of *Torilis japonica* extract (TJE) and reference compounds. Representative chromatograms of TJE, Torilin, and osthol were obtained under identical chromatographic conditions and monitored at 254 nm. Torilin and osthol were used as reference standards for phytochemical characterization of the extract. Several characteristic peaks were detected in TJE, including a major peak (A) and a torilin-associated peak identified by comparison with the authenticated standard. The chromatographic profile provides a characteristic fingerprint of TJE and may serve as a useful tool for extract characterization, quality control, and batch-to-batch reproducibility assessment. The numerical labels above the major peaks indicate the retention times (min) of the corresponding chromatographic peaks (TJE: 28.889 min, torilin: 28.877 min, and osthol: 27.800 min), which are provided as reference values for peak identification. Modified and adapted from Seo et al. [30]. Peak A was additionally indicated in the present study to facilitate interpretation of the characteristic chromatographic profile of TJE.

To further characterize the phytochemical composition of *Torilis japonica* extract (TJE), high-performance liquid chromatography (HPLC) analysis was performed using torilin and osthol as reference compounds. The HPLC chromatogram shown in Figure 1 was adapted from our previous study [30] and is presented here to describe the characteristic phytochemical profile of TJE used as the base extract for the present study. Representative chromatograms of TJE and the corresponding standards are presented in Figure 1. The HPLC chromatogram of TJE revealed several characteristic peaks between approximately 25 and 31 min, including a prominent peak designated as Peak A. Comparison with reference standards demonstrated the presence of a torilin-associated peak, confirming torilin as a characteristic marker compound of TJE. The overall chromatographic profile provides a reproducible fingerprint for extract characterization and may serve as a useful tool for quality control and batch-to-batch consistency assessment.

### 3.2. TJE Suppresses AD-Associated Biomarkers in HaCaT Cells

**Figure 2:**
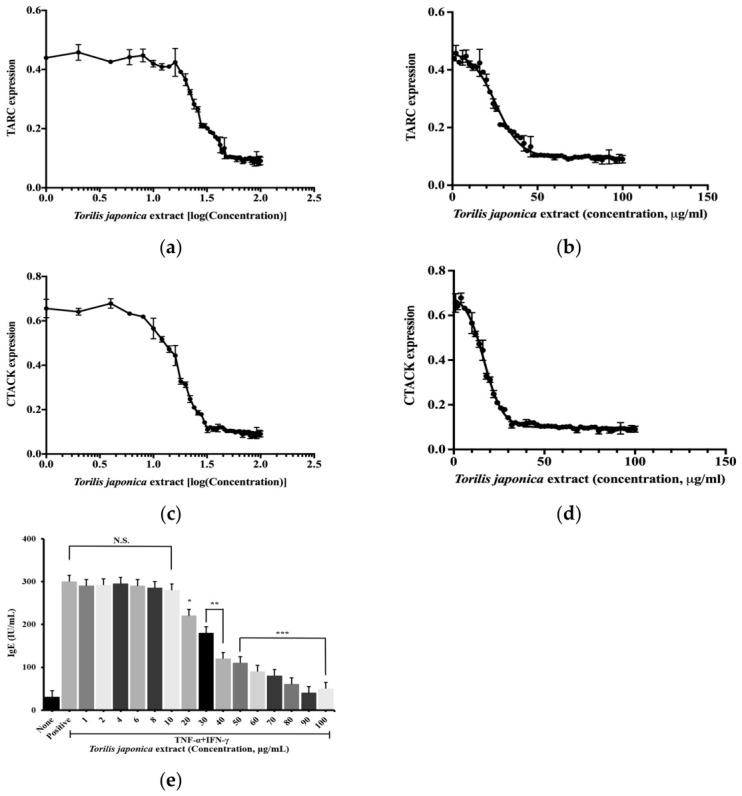
AD-related biomarker levels in TJE-treated HaCaT cells under TNF-α/IFN-γ-induced inflammatory conditions. (**a**) Dose–response curve for TARC levels against log-transformed TJE concentrations. (**b**) TARC levels in response to increasing TJE concentrations (μg/mL). (**c**) Dose–response curve for CTACK levels. (**d**) CTACK levels following treatment with increasing concentrations of TJE. (**e**) IgE levels (IU/mL) across treatment groups, including untreated and TNF-α/IFN-γ-treated positive controls. AD-like inflammatory conditions were induced by pretreatment with TNF-α/IFN-γ, followed by exposure to TJE. Cell culture supernatants were collected 24 h after treatment, and biomarker levels (TARC, CTACK, and IgE) were quantified using ELISA. Data are presented as mean ± SD from technical triplicates (*n* = 3). Statistical significance was analyzed using one-way ANOVA followed by Tukey’s multiple-comparison test. N.S., not significant. * *p* < 0.05, ** *p* < 0.01, and *** *p* < 0.001 versus the TNF-α/IFN-γ-treated positive control group.

The impact of TJE on AD-associated biomarkers was evaluated using a TNF-α/IFN-γ-stimulated HaCaT cell model. Exposure to TNF-α/IFN-γ markedly increased the secretion of TARC, CTACK, and IgE, confirming the establishment of an AD-like inflammatory condition. Compared with the TNF-α/IFN-γ-treated positive control group, TJE treatment significantly reduced the levels of these biomarkers in a concentration-dependent manner (Figure 2). Peptide-only treatment also decreased biomarker expression, although the inhibitory effect was less consistent than that observed with TJE. TJE exhibited potent inhibitory activity, with IC_50_ values ranging from 24.76 to 26.4 μg/mL for TARC and from 16.24 to 17.19 μg/mL for CTACK. The simultaneous suppression of TARC, CTACK, and IgE suggests that TJE effectively attenuates Th2-associated inflammatory responses in HaCaT cells, even at relatively low concentrations.

### 3.3. Optimization of the TJE–GHK-Cu Complex Ratio

**Figure 3:**
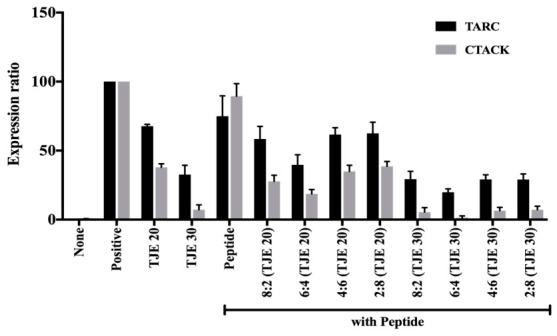
Optimization of the TJE–GHK-Cu complex ratio. HaCaT cells under TNF-α/IFN-γ-induced AD-like inflammatory conditions were treated with TJE alone at 20 μg/mL (TJE20) or 30 μg/mL (TJE30), or with TJE–GHK-Cu complexes prepared at varying TJE:GHK-Cu weight ratios (8:2, 6:4, 4:6, and 2:8, *w*/*w*). Treatment with TJE alone or TJE–GHK-Cu complexes reduced AD-associated biomarker levels relative to the TNF-α/IFN-γ-treated positive control group. The 6:4 TJE:GHK-Cu formulation showed the most pronounced overall reduction in inflammatory biomarkers and was therefore selected as the optimized formulation for subsequent experiments. Data are presented as mean ± SD from technical triplicates (*n* = 3).

To identify an effective formulation, HaCaT cells under AD-like conditions were treated with varying ratios of TJE and GHK-Cu (8:2, 6:4, 4:6, and 2:8; TJE:GHK-Cu (*w*/*w*)). Compared with the TNF-α/IFN-γ-treated positive control group, the combined formulations reduced TARC and CTACK levels to varying degrees. Among the tested ratios, the 6:4 formulation showed the most pronounced overall reduction in these biomarkers (Figure 3). Based on these readouts, the 6:4 ratio was selected as the optimized formulation for subsequent experiments.

### 3.4. TJE–GHK-Cu Complex Downregulates Th2 Cytokine Expression at the Transcriptional Level

**Figure 4:**
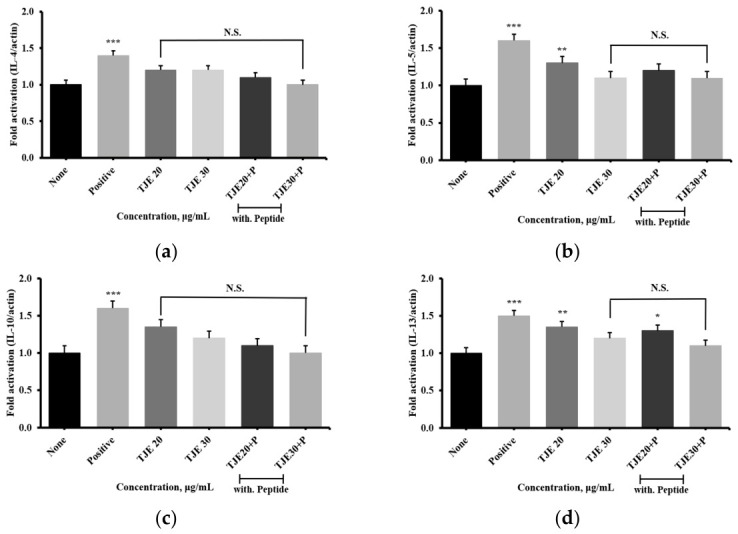
Effects of TJE and the TJE–GHK-Cu complex on Th2 cytokine gene expression in TNF-α/IFN-γ-induced HaCaT cells. Relative mRNA expression levels of (**a**) IL-4, (**b**) IL-5, (**c**) IL-10, and (**d**) IL-13 were quantified by RT-qPCR and normalized to β-actin. None, PBS-treated negative control; Positive, TNF-α/IFN-γ-treated positive control; TJE20 and TJE30, TJE-treated groups (20 and 30 μg/mL, respectively); TJE20+P and TJE30+P, TJE–GHK-Cu complex-treated groups. AD-like inflammatory conditions were induced by TNF-α/IFN-γ pretreatment prior to sample exposure. Data are presented as mean ± SD from technical triplicates (*n* = 3). Statistical significance was analyzed using one-way ANOVA followed by Tukey’s multiple-comparison test. N.S., not significant; * *p* < 0.05, ** *p* < 0.01, and *** *p* < 0.001 compared with the negative control (None) group, unless otherwise indicated by brackets.

The effects of TJE and the TJE–GHK-Cu complex on Th2-associated cytokine expression were evaluated in TNF-α/IFN-γ-stimulated HaCaT cells using qPCR analysis. AD induction markedly increased the mRNA expression levels of IL-4, IL-5, IL-10, and IL-13 compared with the negative control group, confirming the establishment of an AD-like inflammatory condition (Figure 4). Treatment with TJE reduced the expression of these cytokines in a concentration-dependent manner. Co-treatment with peptide generally resulted in a greater reduction in cytokine expression than TJE alone at the corresponding concentrations, although the magnitude of the effect varied among cytokines. Overall, the TJE–GHK-Cu complex exhibited a stronger inhibitory tendency toward Th2 cytokine expression, suggesting that the combined formulation may contribute to the attenuation of AD-associated inflammatory responses at the transcriptional level.

### 3.5. TJE–GHK-Cu Complex Enhances Keratinocyte-Mediated Wound Healing

**Figure 5:**
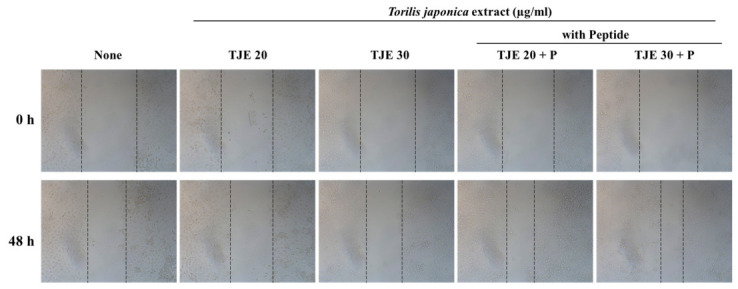
Wound healing progression in HaCaT cells. Following AD induction, a uniform scratch was applied to confluent cell monolayers. Treatments were administered, and wound closure was documented at 0 h and 48 h. The TJE–GHK-Cu complex accelerated cell migration and area reduction significantly better than individual treatments, highlighting an enhanced regenerative capacity.

A scratch assay was used to measure tissue regeneration during AD-like inflammation. The TNF-α/IFN-γ environment significantly delayed wound closure compared to baseline conditions. Applying TJE alone improved cell migration and partially closed the gap. However, the TJE–GHK-Cu complex accelerated the closure rate far beyond the TJE-only treatment (Figure 5). This confirms that blending TJE with GHK-Cu not only mitigates inflammation but directly aids the physical repair of the keratinocyte layer.

### 3.6. Cytotoxicity Assessment Confirms the Safety of the TJE–GHK-Cu Complex

**Figure 6:**
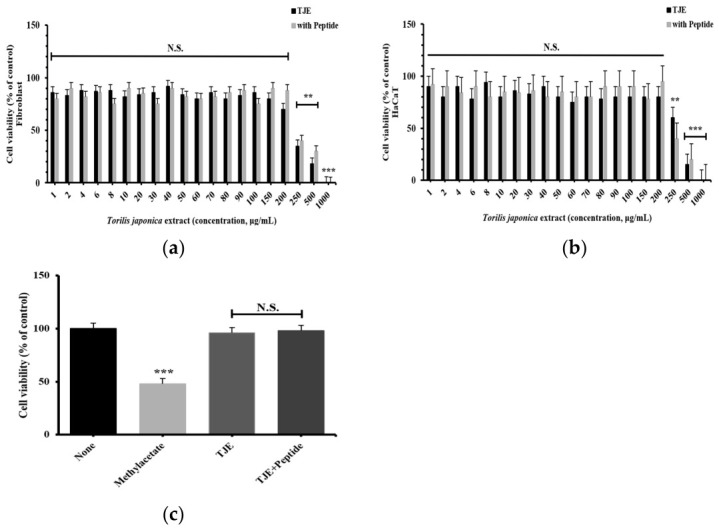
Cytotoxicity assessment of TJE and the TJE–GHK-Cu complex in human fibroblasts and HaCaT keratinocytes. (**a**) Cell viability of human fibroblasts following treatment with increasing concentrations of TJE alone or TJE–GHK-Cu complex, as determined by the MTT assay. (**b**) Cell viability of HaCaT cells following treatment with increasing concentrations of TJE alone or TJE–GHK-Cu complex. (**c**) Comparison of cell viability among the negative control (None), cytotoxic control (methyl acetate), TJE-treated group, and TJE–GHK-Cu complex-treated group. Cell viability was expressed as a percentage relative to the negative control, which was set to 100%. Data are presented as mean ± SD from technical triplicates (*n* = 3). Statistical significance was analyzed using one-way ANOVA followed by Tukey’s multiple-comparison test. N.S., not significant; ** *p* < 0.01 and *** *p* < 0.001, as indicated in each panel.

The cytotoxicity of TJE and the TJE–GHK-Cu complex was evaluated in human fibroblasts and HaCaT keratinocytes using the MTT assay. Cell viability remained above 80% at concentrations up to 200 μg/mL in both cell types, indicating the absence of significant cytotoxicity within the effective concentration range (Figure 6a,b). A reduction in cell viability was observed only at substantially higher concentrations (≥250 μg/mL).

To further assess the safety of the combined formulation, cell viability was compared among the negative control, cytotoxic control (methyl acetate), TJE, and TJE–GHK-Cu complex groups (Figure 6c). While methyl acetate significantly reduced cell viability, both TJE and the TJE–GHK-Cu complex maintained viability levels comparable to the negative control group. The TJE–GHK-Cu complex exhibited approximately 94% cell viability, demonstrating good cellular compatibility under the experimental conditions. These findings indicate that the observed biological effects of TJE and the TJE–GHK-Cu complex are unlikely to be attributable to cytotoxicity.

### 3.7. Antioxidant Activity of the TJE–GHK-Cu Complex

**Figure 7:**
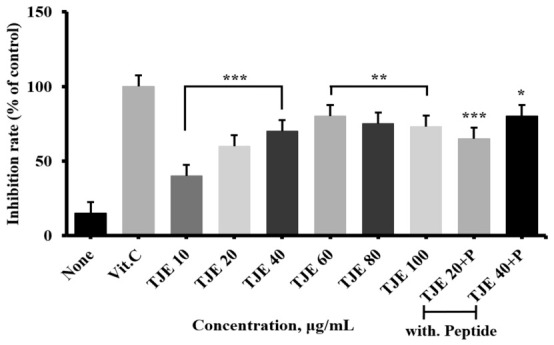
DPPH radical scavenging activity of TJE and the TJE–GHK-Cu complex. Antioxidant activity was evaluated using the DPPH radical scavenging assay. None represents the PBS-treated negative control, and Vit. C represents the vitamin C-treated positive control. TJE10–TJE100 indicate TJE-treated groups at the specified concentrations (μg/mL), whereas TJE20+P and TJE40+P indicate TJE–GHK-Cu complex-treated groups. Radical scavenging activity is expressed as a percentage relative to the vitamin C positive control. TJE exhibited a concentration-dependent increase in radical scavenging activity, reaching approximately 80% of the vitamin C control at 60 μg/mL. Data are presented as mean ± SD from technical triplicates (*n* = 3). Statistical significance was analyzed using one-way ANOVA followed by Tukey’s multiple-comparison test. * *p* < 0.05, ** *p* < 0.01, and *** *p* < 0.001, as indicated in the panel.

The antioxidant activity of TJE and the TJE–GHK-Cu complex was evaluated using the DPPH radical scavenging assay. TJE exhibited a concentration-dependent increase in radical scavenging activity, indicating its ability to donate electrons or hydrogen atoms and neutralize free radicals (Figure 7). At 60 μg/mL, TJE achieved approximately 80% of the radical scavenging activity observed for the vitamin C positive control. Furthermore, treatment with the TJE–GHK-Cu complex resulted in higher radical scavenging activity than TJE alone at the corresponding concentrations. These findings suggest that peptide supplementation may enhance the antioxidant capacity of TJE. The DPPH assay reflects a classical radical-scavenging or chain-breaking antioxidant mechanism, rather than direct suppression of intracellular ROS generation. Given the contribution of oxidative stress to AD-associated inflammation, this improved radical scavenging activity may contribute to the biological efficacy of the TJE–GHK-Cu complex.

## 4. Discussion

AD is a highly complex skin condition shaped by immune system imbalances, physical barrier defects, unchecked oxidative stress, and poor tissue repair [1,2,3,4,5]. Oxidative stress acts as a major trigger in this cycle, driving inflammatory signals and damaging the skin barrier [3,4]. High levels of reactive oxygen species (ROS) not only fuel Th2-driven immune reactions but also disrupt normal keratinocyte behavior and block the production of essential structural proteins, worsening the disease [3,7].

Because AD involves so many overlapping issues, treatments that focus on just one pathway usually fall short of providing long-lasting relief. Effective management requires a broader strategy that simultaneously handles inflammation, oxidative damage, and tissue regeneration. To address this, we developed a combined treatment using TJE—a botanical agent known for its anti-inflammatory and antioxidant traits—and a bioactive GHK-Cu recognized for its ability to modulate cell signaling and promote tissue repair.

Current therapeutic approaches for atopic dermatitis (AD), including topical corticosteroids, calcineurin inhibitors, Janus kinase (JAK) inhibitors, and biologic agents, primarily focus on suppressing inflammatory responses and controlling disease symptoms [3,11]. Although these therapies provide substantial clinical benefits, limitations such as adverse effects associated with long-term use, incomplete restoration of skin barrier function, and variable patient responses remain important challenges [3,4,5,13]. Consequently, increasing attention has been directed toward complementary approaches based on plant-derived bioactive compounds and peptide-based therapeutics [12,14,15,16,17,18,19]. Several plant extracts have demonstrated anti-inflammatory and antioxidant activities in experimental AD models [14,15,16,29], while bioactive peptides have shown potential to promote tissue repair and modulate inflammatory signaling [12,18,19]. In this context, the TJE–GHK-Cu complex was designed to combine the antioxidant and anti-inflammatory properties of a botanical extract with the regenerative functions of a bioactive peptide. The present findings suggest that this multifunctional approach warrants further investigation alongside existing therapeutic strategies for AD.

The fact that TJE suppresses specific AD-related chemokines indicates it directly interferes with the core mechanisms of Th2 inflammation [2,3]. Lower levels of TARC and CTACK mean fewer Th2 cells are drawn to the inflamed skin, cutting off a crucial step in the allergic response [31]. This suppression is likely tied to TJE’s antioxidant capabilities. Oxidative stress activates redox-sensitive pathways NF-κB, which control inflammatory gene expression [3,9,27]. By reducing oxidative stress, TJE indirectly calms these inflammatory pathways while also providing direct anti-inflammatory action. The drop in IgE levels also shows that TJE disrupts upstream Th2 immune signaling [2,11]. These results align with previous research on coumarin derivatives like osthole and imperatorin, which block inflammatory signals by interfering NF-κB and MAPK activation [9,27]. Our findings build on this knowledge, showing that TJE coordinates the downregulation of several key mediators driving AD.

Even with strong anti-inflammatory properties, plant extracts often have limited clinical applicability because of insufficient skin penetration and limited delivery to deeper target sites [17,24]. In this study, GHK-Cu was incorporated into the formulation to complement these limitations and provide additional regenerative activity. This bioactive peptide is well-documented for its role in cellular communication and tissue repair [18,21]. In the present study, the TJE–GHK-Cu complex, particularly at the 6:4 mixing ratio (TJE: GHK-Cu (*w*/*w*)), showed greater inhibitory effects on inflammatory biomarkers than the single-agent treatments, suggesting a potential cooperative or complementary effect [19]. This effect may occur because the components affect different aspects of the disease process; TJE neutralizes free radicals and dampens inflammation, whereas GHK-Cu may contribute to redox modulation through metal ion interactions and support cellular repair [21,32].

This cooperative dynamic suggests that the combined formulation may influence oxidative stress-associated inflammatory responses and downstream inflammatory signaling. At the gene expression level, the TJE–GHK-Cu complex reduced the transcription of Th2 cytokines such as IL-4, IL-5, IL-10, and IL-13, suggesting that it may modulate inflammatory responses at the transcriptional level [2,20]. Since these cytokines contribute to IgE class switching, eosinophil activation, and barrier impairment, their suppression may represent an important interruption of the AD-associated inflammatory cycle. Based on previous reports demonstrating that natural products and bioactive compounds can modulate NF-κB and STAT6 signaling pathways in AD-related inflammatory responses, the inhibitory effects observed in the present study may be associated with these pathways [9,15]. However, the involvement of NF-κB and STAT6 was not directly examined in this study and therefore remains to be verified experimentally. The superior performance of the TJE–GHK-Cu complex compared with TJE alone suggests that peptide supplementation may enhance the regulation of cytokine expression; however, the underlying molecular mechanisms require further investigation.

Promoting keratinocyte recovery and supporting epidermal repair-related responses are important complementary strategies alongside the suppression of inflammation in AD. In the present study, the enhanced cell migration and wound closure observed with the TJE–GHK-Cu complex suggest that the combined formulation may support keratinocyte recovery under inflammatory conditions [13]. GHK-Cu has been reported to contribute to extracellular matrix remodeling and growth factor-related repair processes [21,22,23,25]. Therefore, by combining the protective effects of TJE with the repair-associated properties of GHK-Cu, the TJE–GHK-Cu complex may contribute to keratinocyte recovery beyond the suppression of inflammatory signaling. However, because barrier-associated markers such as filaggrin, loricrin, and tight-junction proteins were not directly evaluated in this study, these findings should be interpreted as evidence of keratinocyte recovery-related activity rather than direct proof of epidermal barrier restoration.

Oxidative stress modulation may also contribute to the biological effects observed in this study [3,16]. The stronger radical-scavenging capacity of the combined treatment suggests that peptide supplementation may provide additional redox-modulating effects or help stabilize the antioxidant activity of TJE [16,32]. The combination of anti-inflammatory and antioxidant properties may contribute to the broad biological activity observed in this study. Importantly, the formulation showed no significant cytotoxicity during the assays, indicating that the observed reductions in inflammatory markers were unlikely to be secondary effects of cytotoxicity. The absence of significant cytotoxicity supports the suitability of the formulation for further preclinical investigation.

From a mechanistic antioxidant perspective, the present findings should be interpreted with distinction between classical radical scavenging activity and preventive antioxidant activity. The DPPH assay demonstrates the ability of TJE and the TJE–GHK-Cu complex to neutralize pre-existing radicals, corresponding to a classical chain-breaking antioxidant mechanism. By contrast, preventive antioxidants are defined by their ability to prevent the initiation of oxidative chain reactions or suppress ROS generation before oxidative damage is propagated. In the TNF-α/IFN-γ-stimulated HaCaT model, the reduction in AD-associated chemokines and Th2-related cytokines may be consistent with attenuation of ROS-linked, redox-sensitive inflammatory signaling. However, because intracellular ROS production was not directly quantified in this study, the classification of TJE and the TJE–GHK-Cu complex as preventive antioxidants should be regarded as a mechanistic possibility rather than a definitive conclusion. Future studies measuring intracellular ROS generation, lipid peroxidation, and redox-sensitive signaling pathways will be necessary to confirm whether these materials exert preventive antioxidant activity in keratinocytes.

Despite these encouraging findings, several limitations should be acknowledged. First, the present study relied on a TNF-α/IFN-γ-stimulated HaCaT keratinocyte model, which is widely used for investigating AD-associated inflammatory responses. However, HaCaT cells cannot fully reproduce the complex immune microenvironment of AD because they lack interactions with immune cell populations such as T lymphocytes, dendritic cells, and Langerhans cells that contribute significantly to disease pathogenesis [1,2,3,4]. Therefore, although this model is useful for evaluating keratinocyte-mediated inflammatory responses, it does not completely reflect the biological complexity of AD observed in patients. Because this study was conducted entirely using in vitro models, the findings cannot fully replicate the systemic immune interactions involved in AD in vivo [3,11]. The exact molecular mechanisms driving the observed effect also require deeper investigation, particularly through direct measurement of intracellular ROS levels, lipid peroxidation, and redox-sensitive signaling pathways. Because the present study assessed radical scavenging activity using the DPPH assay but did not directly quantify ROS generation in stimulated keratinocytes, the potential classification of TJE and the TJE–GHK-Cu complex as preventive antioxidants remains to be confirmed experimentally. We also have yet to evaluate the long-term stability, pharmacokinetic profile, and exact skin permeability of the TJE–GHK-Cu complex. Future studies employing more physiologically relevant systems, including three-dimensional skin models and in vivo animal models of AD, are warranted to further validate the therapeutic efficacy and mechanism of action of the TJE–GHK-Cu complex. Additional mechanistic studies are required to determine whether NF-κB, MAPK, and STAT6 signaling pathways contribute to the biological effects of the TJE–GHK-Cu complex. Furthermore, the present study did not directly evaluate barrier-associated proteins such as filaggrin, loricrin, or tight-junction markers. Future studies incorporating Western blotting, immunofluorescence analysis, and barrier-related gene expression assays will be necessary to verify these mechanisms. To maximize clinical utility, future work might explore advanced delivery vehicles like hydrogels or nanoparticle carriers to optimize skin penetration and stability [19]. Ultimately, rigorous clinical trials will be needed to verify both safety and efficacy in human patients.

Overall, our findings suggest that the TJE–GHK-Cu complex exerts synergistic anti-inflammatory, antioxidant, and regenerative effects in an in vitro model of AD. By providing anti-inflammatory, antioxidant, and regenerative activities, the TJE–GHK-Cu complex may contribute to the management of multiple pathological features associated with AD. This combination strategy provides a basis for further investigation of multifunctional approaches for chronic inflammatory skin conditions.

## 5. Conclusions

In conclusion, this study highlights the potential anti-atopic activity under the present in vitro conditions of the TJE–GHK-Cu complex, which may affect the intertwined issues of Th2 inflammation, oxidative stress, and keratinocyte damage underlying AD. Combining the botanical extract and the bioactive peptide at a 6:4 ratio (TJE:GHK-Cu (*w*/*w*)) showed greater effects than either component alone, showing stronger responses than those of either component used alone. The antioxidant properties of TJE combined with the regenerative properties of GHK-Cu produced a multifunctional formulation with anti-inflammatory and wound-healing potential. The TJE–GHK-Cu complex maintained high cell viability at the concentrations tested in this study, indicating low in vitro cytotoxicity. These findings support further investigation of TJE–GHK-Cu complex as a potential strategy for addressing multiple pathological features of AD. Moving forward, in vivo testing and clinical trials will be the next steps to evaluate translational relevance.

## 6. Patents

The TJE–GHK-Cu complex formulation investigated in this study is associated with Korean Patent Registration No. KR10-1964249. Prof. Young-Min Kim is an inventor of the patented technology and is affiliated with the organization holding the intellectual property rights related to this formulation. Prof. Kim also serves as the Chief Executive Officer (CEO) of the company associated with the patent. The patented formulation constitutes the basis of the material evaluated in the present study.

## Figures and Tables

**Table 1:** TJE HPLC analysis conditions.

HPLC Condition	
HPLC	Agilent 1200 series
Column	YMC J’sphere ODS-H80
Detector	UV (254 nm)
Flow	1 mL/min
Mobile phase (A: 0.05% TFA in Water, B: MeOH)	80:20 (0–30 min), 0:100 (300 min)

**Table 2:** Primer sequences and PCR conditions for IL-4, Il-5, IL-10, and IL-13.

	Primer Sequence	NCBI Accession Number	Amplification Size (bp)	Annealing Temperature (°C)
IL-4	F: 5′- GCTCCAGACAACCTGGGATA-3′	NM_000589	185	59.5
	R: 5′- TCAGTGAGGAGAGGCTGGTT-3′			
IL-5	F: 5′- GAAGGATGGACCAGGCAGTA-3′	NM_000879	165	60
	R: 5′- GCCTCACCCATCAATCTGTT-3′			
IL-10	F: 5′- CAACTCATGCGTGAGAT-3′	NM_000572	203	60.5
	R: 5′- TACTTGGTGGGGTTTTCGAG-3′			
IL-13	F: 5′- TTTTGTACTGCCTGCTGTGG-3′	NM_002188	151	60
	R: 5′- CAGGAACCTCTGCCTGAAAG-3′			
β-actin	F: 5′-CTGGCACCCAGCACAATG-3′	NM_001101	69	60
	R: 5′-GCCGATCCACACGGAGTACT-3′			

## Data Availability

All data generated or analyzed during this study are included in this published article.

## Abbreviations

The following abbreviations are used in this manuscript:

AD	*Atopic dermatitis*
TJ	*Torilis japonica*
TJE	*Torilis japonica* extract
GHK-Cu	*Glycyl-L-histidyl-L-lysine copper(II) complex*
CCL	*CC chemokine ligand*
TARC	*Thymus and activation-regulated chemokine (CCL17)*
CTACK	*Cutaneous T-cell attracting chemokine (CCL27)*
IL	*Interleukin*
IgE	*Immunoglobulin E*
Th2	*T helper type 2*
TSLP	*Thymic stromal lymphopoietin*
ROS	*Reactive oxygen species*
DMEM	*Dulbecco’s Modified Eagle’s Medium*
FBS	*Fetal bovine serum*
PBS	*Phosphate-buffered saline*
DMSO	*Dimethyl sulfoxide*
TNF-α	*Tumor necrosis factor-α*
IFN-γ	*Interferon-γ*
MTT	*3-(4,5-Dimethylthiazol-2-yl)-2,5-diphenyltetrazolium bromide*
DPPH	*2,2-Diphenyl-1-picrylhydrazyl*
ELISA	*Enzyme-linked immunosorbent assay*
cDNA	*Complementary DNA*
PCR	*Polymerase chain reaction*
qPCR	*Quantitative polymerase chain reaction*
HPLC	*High-performance liquid chromatography*
SD	*Standard deviation*
IC_50_	*Half maximal inhibitory concentration*
STAT	*Signal transducer and activator of transcription*
NF-κB	*Nuclear factor kappa B*
DNCB	*2,4-Dinitrochlorobenzene*
MAPK	*Mitogen-activated protein kinase*
